# A (Dis-)information Theory of Revealed and Unrevealed Preferences: Emerging Deception and Skepticism via Theory of Mind

**DOI:** 10.1162/opmi_a_00097

**Published:** 2023-08-20

**Authors:** Nitay Alon, Lion Schulz, Jeffrey S. Rosenschein, Peter Dayan

**Affiliations:** Department of Computer Science, The Hebrew University of Jerusalem, Jerusalem, Israel; Department of Computational Neuroscience, Max Planck Institute for Biological Cybernetics, Tübingen, Germany; Department of Computer Science, University of Tübingen, Tübingen, Germany

**Keywords:** theory of mind, deception, communication, skepticism, disinformation, multi-agent-RL, IPOMDP

## Abstract

In complex situations involving communication, agents might attempt to mask their intentions, exploiting Shannon’s theory of information as a theory of misinformation. Here, we introduce and analyze a simple multiagent reinforcement learning task where a buyer sends signals to a seller via its actions, and in which both agents are endowed with a recursive theory of mind. We show that this theory of mind, coupled with pure reward-maximization, gives rise to agents that selectively distort messages and become skeptical towards one another. Using information theory to analyze these interactions, we show how savvy buyers reduce mutual information between their preferences and actions, and how suspicious sellers learn to reinterpret or discard buyers’ signals in a strategic manner.

## INTRODUCTION

Actions speak louder than words—sometimes enabling us to infer another person’s beliefs and desires. Savvy speakers spin stories to fit their audience, like house buyers feigning disinterest to get a better deal. In turn, savvy listeners retaliate by ignoring them, like sellers sticking to their original prices. Here, we introduce a minimal two-agent task that captures the essence of such interactions and model agents’ interaction using the reinforcement learning (RL) framework of Interactive Partially Observable Markov Decision Processes (IPOMDP; Gmytrasiewicz & Doshi, 2005), that endows agents with a theory of mind (ToM). We employ information theory to analyze the signalling behavior arising in this novel paradigm, showing how purely reward-maximizing agents endowed with a theory of mind distort and re-interpret signals.

### Theory of Mind as Inverse Reinforcement Learning

In machine learning, inferring an agent’s preferences, or utility function, is part and parcel of models that can be broadly summarized as ‘inverse reinforcement learning’ (IRL; Ng & Russell, 2000). These models observe an agent’s actions and try to deduce the agent’s preferences from these observations. The core insight of these models is that agents will only perform actions that are worth the cost. For example, if your colleague walks three blocks to a fancy cafe when there is a free coffee machine in the office, they likely award high subjective utility to artisanal roasts. Algorithmic methods of exact or approximate Bayesian inference (Baker et al., 2011), including neural network distillations, can be used to carry out IRL (Oguntola et al., 2021; Rabinowitz et al., 2018).

In cognitive science, inverse reinforcement learning has been used for models of the powerful inferences humans draw about one another (Baker et al., 2017; Jara-Ettinger, 2019). Chiefly among them, the ‘Naïve Utility Calculus’ (Jara-Ettinger et al., 2016) proposes that humans reason in ways similar to Bayesian inference. This type of model has successfully explained how we reason about one another’s kindness, knowledge, effort allocation, and skills (Berke & Jara-Ettinger, 2021; Xiang et al., 2023). Interestingly, this inferential ability arises early in development, with children as young as 5 years old being able to make intricate inferences (Jara-Ettinger et al., 2015). Across fields, this ability to reason about others beliefs, desires and intentions has been referred to as ’theory of mind‘ (Premack & Woodruff, 1978), a trait hypothesized to be foundational to the human ability to carry out complex social interactions (Camerer et al., 2015; De Martino et al., 2013; Ho et al., 2022; Hula et al., 2015; Ray et al., 2008; Rusch et al., 2020; Steixner-Kumar et al., 2022; Xiang et al., 2012).

### Extending Inverse Reinforcement Learning Through Recursion

The ‘Naïve Utility Calculus’, however, is not called ‘naïve’ without reason, and its naivety extends to most other ‘naive’ inverse reinforcement learning algorithms: they assume that the agents being observed are acting ‘naively’, that is, in a purely reward maximizing manner. However, in many situations, agents are aware that they are being watched. Such observer awareness (Miura & Zilberstein, 2021) is less important when the observer is an equally coffee-obsessed colleague, but becomes complicated when the observer can use its inferences to our detriment, for example, to increase the price of our favorite espresso—as, for example, in online dynamic pricing. Agents acting optimally should take such competitive scenarios into account when making their decisions—by inferring, and planning with, the inference of the observer in mind.

Such recursive reasoning about other agents extends theory of mind and has been used to explain a number of different phenomena in human interaction, from how we teach to how we lie and wage war (Crawford, 2003; Ho et al., 2022; Oey et al., 2023). Broadly, formal models of this recursivity (Doshi et al., 2020) tend to extend the simple inference in inverse reinforcement learning, and ‘plan through’ this inference model. A particularly flexible instantiation of this is the mulitagent reinforcement learning framework involving Interactive Partially Observable Markov Decision Processes (IPOMDP; Gmytrasiewicz & Doshi, 2005). In an IPOMDP characterization, agents reason not only about uncertainty in the environment (as in a regular POMDP), but also about other agents’ beliefs, desires, and intentions. Furthermore, agents do so in a recursive manner, at different levels of a ‘cognitive hierarchy’ (Camerer et al., 2004). That is, agents of different sophistication model agents that are less sophisticated than themselves (‘I believe what you believe what I believe’ and so on). On average, humans are hypothesized to reason to a depth of 1.6 levels (Camerer et al., 2004).

### Information Theory in Multi-Agent Interactions

In an IPOMDP, one agent’s actions are interpreted by other agents as signals about the hidden or latent characteristics that would be the target of IRL. Information theory (IT) is a particularly helpful tool to understand the messages that thereby get sent among such sophisticated reasoning agents, particularly regarding deception (Kopp et al., 2018; Zaslavsky et al., 2020). This is because it allows us to measure formally the distortion of the signals transmitted between agents, and to pin down the deception and skepticism that possibly arise. Deception thereby wears many hats. One modus operandi is to masquerade, for example faking confidence despite having a bad hand in Poker. Another is to completely disassociate actions from intentions, for example via a Poker face which is the same regardless of the deck.

### Utility Functions in Social Interactions

Previous multiagent reinforcement learning work on shaping other agents has focused on modifying agent’s utility functions to include social aspects, often based on information theory. For example, in Jaques et al. (2019) the utility function of one agent was modified to gain reward from the environment as well as from influencing other agents’ behaviour. Agents endowed with this utility adopted socially aware policies, in which, for example, they learn to signal other agent on the location of an unobserved reward to lead them to otherwise unreachable locations, thus gaining much from the policy change. A similar approach was explored by Strouse et al. (2018). This work is motivated by the observation that in cooperative environments, being transparent about one’s goal creates trust and coordination, while hiding one’s intentions is beneficial in competitive settings. Strouse et al. (2018) handcrafted agents’ utility functions to take such (in-)transparency into account: Specifically, when agents competed, their utility function was augmented with a reward for minimizing the mutual information (MI) between their actions and intentions, whereas when they were cooperated, they were rewarded for maximizing the same term.

The information-theoretic handcrafting approach is in contrast to our approach, which follows the ‘reward is enough’ tradition set by Silver et al. (2021). ‘Reward is enough’ argues that complex behaviours, particularly those observed in the social realm, do not require any hand-crafting. Instead, these behaviours arise in the actions of agents that purely maximize rewards. To preview our findings, we show that this is indeed the case for deception and skepticism. We thereby crucially differ from Jaques et al. (2019) and Strouse et al. (2018) in using information theory only to measure the effects of this reward maximization but not have it be a part of the reward maximization itself. We also note that both papers, while conceptually employing ToM, do so at a shallow level, usually only endowing one agent in the environment with it, and so precluding any true recursivity.

### Deception in AI

Deceptive behaviour in AI has received considerable attention, with increased interest in applications such as deceptive path planning (Masters & Sardina, 2017), military drone control (Ramirez et al., 2018) or resilience to cyber attacks (Rowe, 2003), and with a multi-agent reinforcement learning perspective (Chelarescu, 2021; Sarkadi et al., 2019). Most of this work however rests on *model-free* behaviors, where an agent learns through experience and trial-and-error to deceive. This is for example evident in ruses learnt by systems like AlphaGo (Silver et al., 2016), which uses MCTS for *Q*-value estimation but uses self-play to simulate opponent’s move rather than having an explicit model of the opponent. Similarly, learning to strategically hide information, or at least not fully reveal it, can emerge despite the lack of an explicit model of the opponent, for example in the gameplay of ‘Cicero’ in Diplomacy (Meta Fundamental AI Research Diplomacy Team et al., 2022). However, such model-free systems are in fact sometimes easily trickable (Wang et al., 2023). Such more model-free behavior is in contrast to *model-based* (Dolan & Dayan, 2013) deception, where agents learn to target the inference processes of others, revoking the naivete of the Naive Utility Calculus. However, existing work in this field (Aitchison et al., 2021; Liu et al., 2021) only does so at low levels of recursions, merely asking how to circumvent a naïve utility calculus but not asking how a not-so-naïve utility calculus might act, or how to act deceptively against such a savvy opponent.

## METHODS

### Paradigm

**Figure 1.**
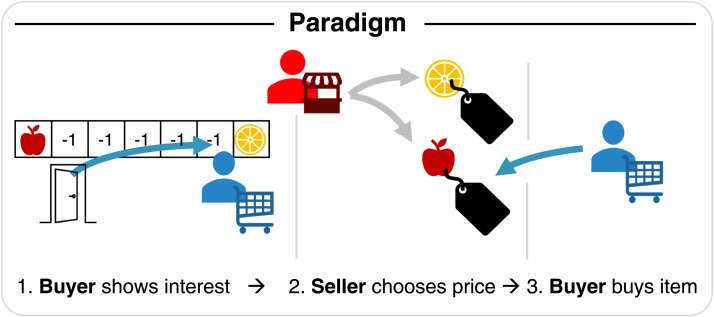
In our simulated paradigm, two agents interact, a buyer (blue) and a seller (red). The buyer first chooses either of two differently rewarding objects, incurring cost on the way to the chosen object via the distance travelled. The seller observes this choice and the accompanying distances, draws inferences about the buyer’s prices, and then prices the items accordingly. Finally, the buyer has to buy one of the items for the set price.

As an example of the emergence of disinformation, we model a buyer and a seller interacting over three periods or stages (Figure 1). Imagine a store owner offering items at stalls in various locations, and using observations of buyer behavior to set prices for subsequent sales to the buyer. We here concern ourselves with a single-shot instance of this task where the buyer and seller only interact once over these three stages, and do *not* learn about each other in a repeated fashion.

In the *first* stage, the buyer enters what can be thought of as a simple T-Maze with two different items located at the ends of opposite arms (in our example, an apple and an orange). The buyer incurs travelling costs, denoted by the distances *d*(apple), *d*(orange), for travelling down one of the arms until it reaches and consumes one of the fruits. It then duly receives a reward based on its preferences. We denote the preferences over items by *r*(apple), *r*(orange), which are the rewards that the agent would receive from consuming them. Thus the buyer’s *immediate* utlity at stage one, *U*_*B,*1_, from an item *i*_1_ (*i*_1_ ∈ {apple, orange}) at this stage is just the reward minus the cost incurred through the distance travelled:

$$U_{B}^{t=1}\left(i_{1},d\right)=r\left(i_{1}\right)-d\left(i_{1}\right)$$ (1)

Crucially, the seller observes the buyer’s decisions (Miura & Zilberstein, 2021). Importantly, the seller is also aware of the buyer’s travelling cost, but is *not* aware of its preferences towards either the apple or the orange, and, as we will see below, needs to infer them.

Approaching this task through an experimentalist’s eyes, we will later vary both the distances of the two objects (*d*(apple), *d*(orange)), as well as the preference the buyer has for them (*r*(apple), *r*(orange)). In essence, these two, distance and preference, make this a bivariate experiment.

In the *second* stage, the seller uses their observation of the buyer to set prices, *m*(*i*_3_), for a future possible purchase of one of these two items. This process requires the seller to infer from the buyer’s selection something about the buyer’s preferences, so that the seller can set prices that maximize the seller’s reward. As mentioned above, this requires a model of the buyer’s behavior. We present these models, and describe the inverse inference process in a later section.

In the *third* stage, the buyer purchases one of the items for the set price, *m*(*i*_3_), and then consumes it, again receiving a reward *r*(*i*_3_) for this consumption according to its preferences:

$$U_{B}^{t=3}\left(i_{3},m\right)=r\left(i_{3}\right)-m\left(i_{3}\right)$$ (2)

The overall (undiscounted) utility accumulated by each agent is thus as follows:

$$U_{B}^{\text{total}}\left(i_{1},i_{3},d,m\right)=U_{B^{1}}\left(i_{1},d\left(i_{1}\right)\right)+U_{B^{3}}\left(i_{3},m\left(i_{3}\right)\right)$$ (3)

The seller’s reward is just the price for the item the buyer buys:

$$U_{S}\left(i_{3}\right)=m\left(i_{3}\right)$$ (4)

Note that *d*(*i*_1_) is environmental (distance to items), while *m*(*i*_3_) is set by the seller. For illustrative purposes, we here restrict the preferences of oranges and apples to sum to 10. We impose the same restriction on the walking distances in the first stage and the prices that the seller can set in the second stage.

### Model and Agents

**Figure 2.**
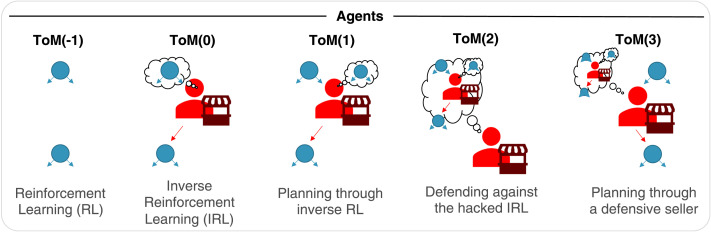
Model: We model agents of different levels of theory of mind (ToM). Note how all buyers and all sellers share the same respective value function and only differ in the way they use theory of mind for planning and/or inference. We begin with a simple reinforcement learning buyer in blue, ToM(−1) that makes the first and second choice independently, and a regular inverse reinforcement learning seller in red, ToM(0), who draws inferences about this buyer. The ToM(1) buyer plans through this seller’s inference process to optimize its overall value. In turn, the ToM(2) seller does inverse reinforcement learning, but of a higher level, taking the ToM(1)’s planning into account. As we will see, this gives rise to a defensive inference process. The ToM(3) in turn plans just like the ToM(1) but does so with the ToM(2)’s ‘defensive’ inference in mind.

We study different levels of sophistication of IPOMDP buyers and sellers arising from the recursive depth of their ToM (see Figure 2). IPOMDPs enable agents to plan through another agent’s inference process via a theory of mind (Figure 1). Unlike Camerer et al. (2015), we assume a strict nesting, where each ToM-level uses only a model of the agent one step below on the ToM ladder as do Gmytrasiewicz and Doshi (2005). Recall that, at the lowest level, this can be understood as planning through another agent’s inverse reinforcement learning (IRL), but can be taken further, allowing ever more sophisticated agents to model one another’s inferences and planning processes recursively.

As the turns in this game alternate, the ToM levels of buyer and seller also alternate (Hula et al., 2015), starting from the simplest buyer, which we denote as ToM(*k* = −1). The **ToM**(−1) **buyer** independently decides based on distance/price and preferences at the first and third stages without taking into account the seller. That is, this naïve buyer solves what amounts to the same utility maximization problem at each step where it gets a choice. As a result, its action-values *Q* at a given state are simply defined as the aforementioned utilities.

$$Q_{k=-1}^{t=1}\left(i_{1},d\left(i_{1}\right)\right)=U_{B}^{t=1}=r\left(i_{1}\right)-d\left(i_{1}\right)$$ (5a)

$$Q_{k=-1}^{t=3}\left(i_{1},d\left(i_{1}\right)\right)=U_{B}^{t=3}=r\left(i_{3}\right)-m\left(i_{3}\right)$$ (5b)

After the buyer computes these *Q*-values, it then selects an item using a SoftMax policy (Equations 6a, 6b) with inverse temperature *β*. If we use *j* to denoting the ‘other’ item (*j* represents ‘apple’ if *i* represents ‘orange’), then the probabilities of the actions *a*_1_ and *a*_3_ of selecting item *i*_1_ and *i*_3_ at stages 1 and 3 are:

$$P_{k=-1}^{t=1}\left(a_{1}=i_{1}|r,d\right)=\sigma \left(\beta \left[Q_{k=-1}^{t=1}\left(i_{1},d\left(i_{1}\right)\right)-Q_{k=-1}^{t=1}\left(j_{1},d\left(j_{1}\right)\right)\right]\right)$$ (6a)

$$P_{k=-1}^{t=3}\left(a_{3}=i_{3}|r,m\right)=\sigma \left(\beta \left[Q_{k=-1}^{t=3}\left(i_{3},d\left(i_{3}\right)\right)-Q_{k=-1}^{t=3}\left(j_{3},d\left(j_{3}\right)\right)\right]\right)$$ (6b)

In RL terms, these action probabilities are referred to as the agent’s policy. Since *r*(*i*) + *r*(*j*) = *d*(*i*) + *d*(*j*) = *m*(*i*) + *m*(*j*), these expressions only depend on one member of the pairs of rewards, distances and prices.

To make inferences about the preferences of the ToM(−1) buyer, the **ToM**(0) **seller** performs Bayesian inverse reinforcement learning (Ramachandran & Amir, 2007) on the buyer’s first stage actions and sets the prices accordingly. The IRL process (Equation 7) inverts the selection using Equation 6a and reweighed using the prior *p*(*r*(*i*_1_)), assumed to be a uniform distribution 𝒰[0, 10] in this paper.

$$p_{k=0}\left(r|a_{1}=i_{1}\right)\propto {\hat{P}}_{k=-1}\left(a_{1}=i_{1}|r,d\right)p\left(r\right)$$ (7)

Here, we note that what the seller assumes to be the buyer’s likelihood, $\hat{P}$_*k*=−1_(*a*_1_ = *i*_1_|***r***, ***d***), in this case is just the ToM(−1)’s policy we defined above in Equation 6a.

Given the posterior beliefs, the ToM(0) seller sets the optimal prices via expected utility maximization. That is, the optimal price of item *i* is set via:

$$\begin{array}{l} m_{k=0}^{*}\left(i_{3},a_{1}\right)=\arg\;\underset{m(i)}{\max}\{E\left[U_{S}(i)|a_{1}\right]\}\\ \;=\arg\;\underset{m(i)}{\max}\left\{\int m(i)\cdot {\hat{P}}_{k=-1}\left(a_{3}=i|r(i),m(i)\right)p_{k=0}\left(r(i)|a_{1}=i_{1}\right)dr(i)\right\} \end{array}$$ (8)

This computation states that for every possible item price *m*(*i*) the seller computes the subjective probability that the ToM(−1) buyer will buy the item at that said price: $\hat{P}$_*k*=−1_(*a*_3_ = *i*|*r*(*i*), *m*(*i*)) using Equation 6b. This *potential* expected revenue: $\hat{U}$_*S*_(*i*) = *m*(*i*) · $\hat{P}$_*k*=−1_(*a*_3_ = *i*|*r*(*i*), *m*(*i*)), is weighted by the posterior probability over the buyer’s preferences *p*_*k*=0_(*r*(*i*)|*a*_1_ = *i*_1_), computed via the inverse reinforcement learning spelled out in Equation 7. Note that while the buyer’s decision-making is stochastic, the seller’s policy sets a deterministic price for each item.

While this has so far been a simple pairing of reinforcement learner and inverse reinforcement learner, agents with higher theory of mind will make more complex decisions.

Specifically, the **ToM**(1) **buyer** takes the ToM(0) price computation into account, and plans through it to optimize the sum of both first and third stage payoffs. Crucially, this model-based planning takes into account the seller’s inverse reinforcement learning, as the expectation in Equation 9 is with respect to this instance of IRL. This planning is computed through a full planning tree span. After the simulation terminates it outputs the action-values (*Q*(*a*)) for each item selection in the first phase. The ToM(1) selects the first action via a SoftMax policy like the ToM(−1) buyer. Since the buyer cannot affect the seller’s behavior in the last step, the ToM(1) acts in an identical way to the ToM(−1), that is, it selects an item via utility maximization.

The key factor in the ToM(1) policy is the belief manipulation of the ToM(0) seller. Since the pricing policy affects the overall utility of the buyer, but is affected by the buyer’s decision in the first step, we can express the *Q*-values of the *k* = 1 buyer at the first stage as:

$$\begin{array}{l} Q_{k=1}^{t=1}\left(a_{1}=i_{1},d\right)=U_{B}^{t=1}\left(i_{1},d\right)+E\left[U_{B}^{t=3}\left(i_{3},m_{k=0}^{*}\left(i_{3},a_{1}\right)\right)|a_{1}=i_{1}\right]\\ \;=U_{B}^{t=1}\left(i_{1},d\right)+\underset{i_{3}}{\sum }U_{B}^{t=3}\left(i_{3},m_{k=0}^{*}\left(i_{3},a_{1}\right)\right)\hat{P}\left(a_{3}=i_{3}|m_{k=0}^{*}\left(i_{3},a_{1}\right),a_{1}=i_{1}\right) \end{array}$$ (9)

Unpacking this equation, the ToM(1) agent performs a thought experiment in which it envisions itself acting as the ToM(0) seller—observing the first item pick and setting the prices accordingly. The first component of this computations is the utility from the buyer’s first selection (Equation 1). Knowing that this selection (*a*_1_) is used by the seller to determine the items’ prices in the next step: $m_{k=0}^{*}$(*i*_3_, *a*_1_), it uses its mental model of the seller to simulate the seller’s optimal pricing process (Equation 8). This nested reasoning includes the seller’s IRL process (Equation 7). Thus, the ToM(1) buyer can predict how its action in the first step affects the potential reward in the last phase through full mentalization of the seller’s inference and learning process.

Crucially, while the ToM(0) seller makes inferences about the ToM(−1) buyer’s utility from behavior, which is the equivalent of IRL, the ToM(1) buyer makes inferences about the optimal pricing of the ToM(0) seller given the buyer’s own selection in the first phase. Note that in the last step of the task the prices are given. Hence, the seller selects an item that maximizes its utility similarly to the ToM(−1) item selection (Equation 6b). Hence, the strategic aspect of the buyer’s planning is the first-item selection, as it is this that affects the beliefs of the seller about the buyer’s preferences.

During planning, the buyer ‘imagines’ its own initial action, and then simulates the seller’s best response as in Equation 8. This simulation performs the hypothetical IRL of the seller and its consequent beliefs, and then computes the optimal price from its policy. Thus, as discussed in the next section, the ToM(1) buyer improves its utility through shaping the beliefs of the ToM(0) seller. The ToM(1) choice at the first stage is selected through a SoftMax policy (similar to the ToM(−1) policy):

$$P_{k=1}^{t=1}\left(a_{1}=i|r,d\right)=\sigma \left(\beta \left[Q_{k=1}^{t=1}\left(i_{3},d\left(i_{3}\right)\right)-Q_{k=1}^{t=1}\left(j_{3},d\left(j_{3}\right)\right)\right]\right)$$ (10)

The **ToM**(2) **seller** models the buyer as a ToM(1) buyer, and is aware of the manipulation schema deployed by it. In essence, it uses the same principles as the ToM(0) seller for its inverse RL, but unlike the ToM(0) seller, it learns to treat the signal provided (item selection) with caution by using the ToM(1)’s policy as its likelihood in the Bayesian update:

$$p_{k=2}\left(r|a=i_{1}\right)\propto {\hat{P}}_{k=1}\left(a=i_{1}|r,d\right)p\left(r\right)$$ (11)

Lastly, the **ToM(3) buyer** again attempts to ‘hack’ the ToM(2) beliefs in a similar manner to the way the ToM(0) attempts to manipulate the ToM(0) seller. This deception depends on the ‘wiggle room’ left, given the defensive policy adopted by the ToM(2) seller.

To hone in on an important point: each agent higher up in the cognitive hierarchy nests the inference and planning of those agents below it in the cognitive hierarchy. Essentially, there is ever more sophisticated reinforcement learning (planning) that gives rise to a policy, and ever more sophisticated inverse reinforcement learning that inverts this policy.

### Information Theory, Deception and Skepticism

As we briefly highlighted in the introduction, information theory presents an elegant tool to analyze the signals sent between agents, and their deceptive as well as skeptical nature. It allows us to do so from different perspectives:

1. From the perspective of the sender of a message, we can ask *how much a message reveals* about something it wants to hide. As we will see, this will be particularly relevant when asking how much the buyer’s actions reveal about its preferences. Information theory allows us to capture this using the Mutual Information between the buyer’s actions and its preferences, *I*(**r**, *a*_1_).
2. From the perspective of the receiver of the message, we can analyse *how much credence is lent to a signal*. We can do so by calculating how much a receiver’s beliefs change in response to a signal. Information Theory lets us do this via the Kullback-Leibler (KL) Divergence between a receiver’s prior and its posterior once it has seen a message. Here, we are interested in this case for the seller before and after it has seen the buyer’s action: *D*_*KL*_(*p*(*r*|*a*_1_)‖*p*(*r*)). Essentially, the lower this divergence, the more skeptical an agent.
3. Finally, we can take a more bird’s eye view of an interaction and ask *how much a given signal sent by a sender is misinterpreted by a receiver*. Again, we can do so using KL-Divergence, for example between what the receiver assumes is a sender’s policy and what the sender’s actual policy is. In our case, we are for example interested in how simpler sellers might be led astray by higher level theory of mind buyers. We measure this as the KL-distance between what a ToM(*k*) seller *assumes* to be the buyer’s policy (i.e., the ToM(*k* − 1) buyer) and the ToM(*k* + 1)’s actual policy, for example for the the case of the ToM(0) buyer: *D*_*KL*_($p_{k=1}^{t=1}$(*a*_1_|*r*, *c*)‖$p_{k=-1}^{t=1}$(*a*_1_|*r*, *c*)). Essentially, this quantifies how effective a sender’s deception is, and is therefore different from the mutual information outlined above, which is more about the mere hiding of information (Kopp et al., 2018).

## RESULTS

We present the agents’ policies resulting from this progressive, recursive modelling. As described above, the only strategic action of the buyer is its first move; hence we compare this action across different ToM levels. In addition, we present the seller’s corresponding prices. We describe how each buyer’s behaviour can be seen as a ‘best-response’ to its perceived opponent. In turn, we discuss how the sellers respond to these policies. Throughout, we quantify these behavioural dynamics with key information theoretic metrics and highlight the relation between the two in light of the cognitive hierarchies.

We describe the intricacies of these policies in substantial detail in order to exploit the simplicity and transparency of our setting.

**Figure 3.**
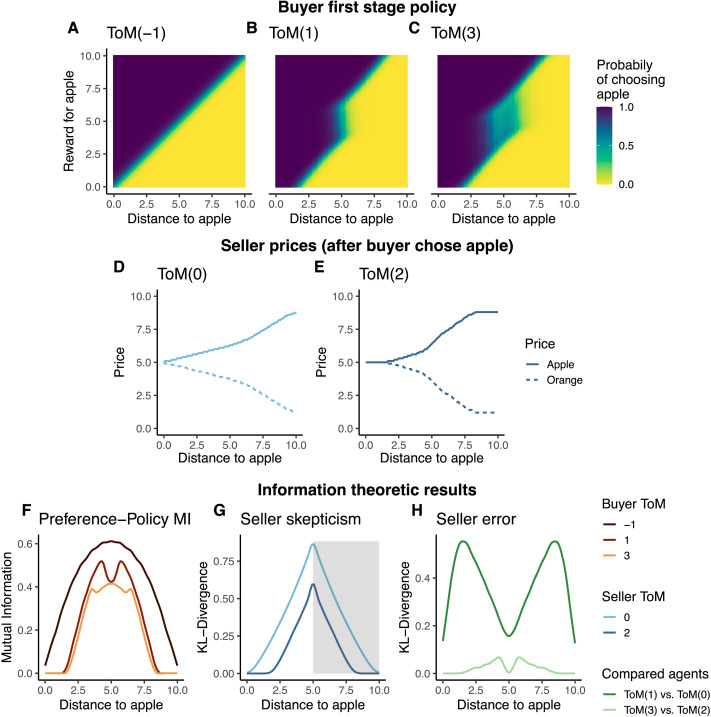
(A–C) Buyer policy in first stage as a function of the distance *d*(apple) and the preference towards the apple, *r*(apple), shown by different ToM-levels. Here, we are using a soft policy with a temperature of *β* = 0.5, as for example in equation 6a. The policies are symmetric because of the constraints. Notice the ruse in ToM(1) and ToM(3), who shift their policies. (D–E) Seller prices, m, after a buyer chose the apple as a function of the distance to the apple, *d*(apple), for the two different seller ToM-levels. ToM(2) discards the evidence at the extremes. (F) Amount of deception by the different ToM buyers quantified by the mutual information between the buyer’s apple preferences and the probability that they will pick an apple. ToM(1) and ToM(3) show lower values, effectively hiding their preferences. (G) Strength of the seller’s belief update quantified by the KL-Divergence between their (flat) prior and posterior over the apple preferences, after observing the buyer choose the closer and thus more likely object (apple in left half, orange in shaded right half). (H) Dissimilarity between the *ToM*(*k*) seller’s assumed policy and the *ToM*(*k* + 1) buyer’s actual policy, simultaneously showing the hacking success of the buyer and error of the seller.

We begin with the **ToM**(−1) **buyer**. As we outlined in the model section, this buyer acts naïvely, maximizing the utility of each stage separately. Figure 3A shows the probabilities of choosing an apple, the x-axis describing the distance from the entrance to the apple, *d*(apple_1_), and the y-axis the reward derived from consuming the apple at the end of the corridor, *r*(apple_1_). The colours represent apple selection probability. Here, due to the symmetric nature of the problem we only discuss the apple selection.

The policy resulting from the ToM(−1)’s value computations are straightforward: The apple is more likely to be chosen when it is closer (left of x-axis) and more preferred (top of y-axis). This behaviour is well aligned with the lack of opponent model—the ToM(−1) buyer does not try to conceal its preferences, since it does not model the seller’s pricing scheme and so believes that its behaviour has no consequences.

As we noted, can re-express such a lack of concealment in information-theoretic terms by measuring how informative the buyer’s choice is about its preferences, i.e., the Mutual Information (MI) between the buyer’s preferences and their initial choice, *I*(**r**, *a*_1_). This MI is shown in the darkest curve in Figure 3F—the action is generally informative, particularly when the fruits are nearly equidistant from the buyer. Here, the buyer’s choice reveals most about its preferences.

The ToM(−1) buyer in turn is the input to **the ToM**(0) **seller**. This seller can translate what it knows about the naïve ToM(−1) policy into prices via simple inverse reinforcement learning. Figure 3D shows these prices for the case that the buyer has chosen the apple in the first stage. Since the ToM(−1) signal is reliable, the distance covered by the buyer is a good proxy for its preferences. For example, if the apple is situated 8 steps from it, and the buyer picks it, then the seller can infer that the buyer’s utility is at least 8 and set the price just shy of this. This all but guarantees that the apple will be selected at the last phase.

Using IT, we re-express this “trust” in the buyer’s action in mathematical terms. Specifically, we measure the strength of the seller’s belief update via the KL-divergence (KLD) between its (flat) prior and posterior, *D*_*KL*_(*p*(*r*|*a*_1_)‖*p*(*r*)). In the lighter curve in Figure 3G, we show the ToM(0) seller’s KLD associated with the choice of the more likely item, which is apple when apple is closer and orange when orange is closer (the latter shown in the shaded area). This highlights how the seller uses every decision of the buyer as a signal, irregardless of the maze set-up.

Aiming to get the best price possible, the **ToM**(1) **buyer** attempts to hack this pricing scheme by playing what amounts to a gambit. This manipulation is manifested in three different ways that are evident in Figure 3B. First, the ToM(1) buyer adopts a deceptive maneuver for the case when the apple is close but *undesired* (the uniformly dark-purple part between *d*(apple) = 0 and *d*(apple) = 2). In this setting, the buyer knows that the ToM(0) seller would interpret an orange selection as a signal for high orange preference. Hence it selects the apple despite its lower appeal to convince the seller that it prefers the apple to orange and gain a lower price for the orange down the road.

The second deception takes place in the shift of location of the indifference line (for example when *d*(apple_1_) = 7). Here, the probability of selecting an apple rises above parity only when the preferences are about 7.5 instead of 7 (the expected behaviour by the observing ToM(0)). This “delayed” selection shifts the posterior beliefs of the ToM(0) seller to believe that the buyer prefers oranges more than it actually does and the buyer gets a discounted price for the apple.

Lastly, in the central region (*d*(apple_1_) = 5) the ToM(0) policy outputs a slightly off-diagonal line of indifference. Again, from the perspective of the ToM(0) observer, the probability of selecting an apple in this setting, when the apple preference is 6.0, are almost 1.0 and not 0.5—thus the naïve ToM(0)’s inverse RL process is inaccurate.

We can express this ruse in information-theoretic terms in two ways. Returning to the MI between (naïve) preferences and policy, we show how the ToM(1) buyer manages to significantly reduce this informativeness about its preferences, particularly when one of the items is close (the medium line in Figure 3F).

As we discussed, we can measure the success of the buyer’s ruse by asking how wrong the ToM(0) seller’s model is. We do so by measuring the KLD between what the ToM(0) seller *assumes* to be the buyer’s policy (i.e., a ToM(−1) buyer) and the ToM(1)’s actual policy, *D*_*KL*_($p_{k=1}^{t=1}$(*a*_1_|*r*, *c*)‖$p_{k=-1}^{t=1}$(*a*_1_|*r*, *c*)). This belief discrepancy is shown in a darker green line in Figure 3H, highlighting the large discrepancies.

Having access to this ToM(1) buyer model, the **ToM**(2) **seller** becomes skeptical about the buyer’s actions, and adjusts its pricing appropriately. We show this pricing in Figure 3E. When the maze setting enables the ToM(1)’s bluff (for example, when the distance to the apple *d*(apple_1_) = 1), observing an apple selection provides the seller with no information about the buyer’s preferences (compare the previously discussed uniformly purple policy of the ToM(1) agent in Figure 3B, and notice how it always chooses the apple regardless of preference). As a result, the seller ignores the distance travelled by the buyer and keeps pricing the items equivalently at these distances.

As the cost of bluffing increases, the seller adapts the apple price to match, but does so at a sub-linear rate, still remaining suspicious of the buyer’s choice—which is warranted by the buyer’s policy of over-selecting the under-preferred item. However, when the apple is farther away than the orange (right half of the plot), this logic switches, and picking the apple now becomes a very strong signal of the buyer actually liking the apple. This is because the ToM(1) buyer is now more likely to employ a similar ruse towards the orange, and would only pick an apple when it has a really strong preference towards it.

Information theory again lets us formalize this skepticism via the KL-divergence as a function of the item more likely to be picked (see the dark line in Figure 3G, which shows how the ToM(2) seller’s belief about the buyer is affected less, or, when the items are closer, not at all, by the buyer’s actions).^1^

The **ToM(3) buyer** attempts to manoeuvre around this skeptical pricing to achieve the best overall reward. However, it is essentially cornered and can only attempt minimal ruses in a few possible game settings, particularly when the items are roughly equidistant (see Figure 3C). In fact, it must act like a ToM(1) buyer because the somewhat paranoid ToM(2) would otherwise overprice the preferred item heavily.

This inability to outmanoeuvre the seller significantly has an information-theoretic consequence. While the Mutual Information between policy and naïve preferences of the ToM(3) buyer is, in some regions, slightly lower than the ToM(1)’s, the ToM(3) buyer cannot mischaracterize its preferences further (lightest curve in Figure 3F). Despite the limited possibilities for deception, this figure shows that through its recursive inference, the ToM(3)’s policy reduces MI, in a way that corresponds to the MI reduction of the ToM(1) agent. When the apple’s distance lies in the interval [3.0, 7.0], the MI between the ToM(3)’s actions and preference displays an opposite pattern relative to the ToM(1)—when the latter’s MI increases, the former’s MI decreases ([3.0, 3.5]) and when the MI of the ToM(1) decreases ([4.0, 5.0]), that of the ToM(3) increases. We interpret this negative correlation through the lenses of the deception of the ToM(3). When the ToM(1)’s MI increases, more information about its preferences leaks through its actions, allowing the ToM(1) seller’s belief update to be more affected by the observed event, as evident in Figure 3G. Through its process of recursive inference, the ToM(3) buyer determines that it has to reduce the MI to avoid preference detection by the ToM(2) seller. In a less constrained environment, this antithetical pattern may be stronger. Equally, the discrepancy between the ToM(2) seller’s assumptions about the ToM(3) buyer and the truth is much less than that for the ToM(0) seller and ToM(1) buyer pair (light curve in Figure 3H). Note that the ToM(2) dissimilarity increases in regions where the ToM(0) dissimilarity decreases, showing the ToM(3)’s attempts at deception.

## DISCUSSION AND FUTURE WORK

Our work shows how purely reward-maximizing agents can appear to engage in complex signalling behavior, as captured by information theory. Unlike Strouse et al. (2018), where the mutual information manipulation is pre-programmed into the utility function of the agents, in our work we only rely on theory of mind and planning. We also extend multi-agent reinforcement learning work on deception (Aitchison et al., 2021; Liu et al., 2021) deeper into the cognitive hierarchy. We show how, through a form of planning that is opponent-aware, agents can exploit other agents’ inference processes. More specifically, we show how a sender can purposefully reduce the informativeness of their actions and target the inferences they expect a receiver to perform. On the other hand, we show how agents can defend themselves from this manipulation by (partially) ignoring the behaviour they observe. This counter-deception can be interpreted as skepticism and we illustrate it both in policies and in inference.

We quantified the extent of manipulation in these deceptive behaviours using information-theoretic metrics. Our results follow the conceptual ideas presented by (Kopp et al., 2018): agents learn to distort the communication by reducing informative information and deliberately convey wrong information. These actions mangle their counterpart’s inference process and cause them to adopt false beliefs. We show how different ToM levels adopt different information-theoretic ‘attacks’.

This work is relevant for the study of social cognition in artificial (Jaques et al., 2019; Rabinowitz et al., 2018) and biological systems. For example, it adds a model-based reinforcement learning and information theoretic perspective to Goodhart’s law (Goodhart, 1984), which states that people tend to try to game statistical regularities used by authorities (e.g., the government or a retailer) for control purposes (e.g., taxation or dynamic pricing). Our agents also engage in rational ‘deliberate ignorance’, or the practice of purposefully discarding signals that they might receive. Deliberate ignorance has received interest as both a description of empirical human behavior and as a potentially useful strategy in game-theory and law (Hertwig & Engel, 2016, 2021). Future research will have to investigate the crucial question of how closely humans (Barnett et al., 2021; Ransom et al., 2017), or other animals (Premack & Woodruff, 1978), actually follow our theoretic analyses in this task.

In general, animals and humans can engage in what appears to be behavior that is similar to what we observe theoretically. For example, Clayton et al. (2007) and Emery and Clayton (2001) observed that corvid engage in sophisticated ruses to hide their food-cache when they are being watched. Of particular interest here is that only those corvids that had previously stolen do so, raising interesting questions about how biological agents learn to rise in the ToM hierarchy.

In humans, ToM-driven deception has also been observed. For example, Crawford (2003) analyzed the Allies’ decision to land in Normandy instead of Calais through the lens of theory of mind. Relevant to our work, Oey et al. (2023) use a recursive modelling framework similar to ours. They show that only a recursive theory of mind style model could explain several empirical facets of how senders design lies. Using less formal models, the developmental psychology literature has investigated how theory of mind may be a precursor for children’s ability to lie: For example Ding et al. (2015) show how teaching kids theory of mind skills led them to ‘deceive constantly’, and in two larger scale meta-analyses Sai et al. (2021) and Lee and Imuta (2021) show a correlation between children’s theory of mind abilities and lying. Seeing that inverse reinforcement learning has been taken as a basis for benevolence about other people in the form of the Naïve Utility Calculus, we believe that IPOMDP, framed as an extension of IRL, is a strong candidate for extending this reasoning systematically to more competitive domains. Thereby, IPOMDP allow for a joint Bayesian reinforcement learning account of social interaction (FeldmanHall & Nassar, 2021; FeldmanHall & Shenhav, 2019). We also suggest that information theory will be a useful tool to understand the messages sent between deceptive and skeptical children, and adults.

### Theory of Mind: Advantages and Limitations

Our work focuses on deep recursion in theory of mind. The benefits of such recursion have been studied in multiple environments, ranging from cooperative (Devaine et al., 2014) to competitive (Hula et al., 2015). While it is well accepted that higher ToM level can improves the outcome of the individual, it is not clear whether this improvement is linear or asymptotic. Our work speaks to this by showing how a first step of deception (ToM(1)) is countered with relatively sweeping skepticism (ToM(2)).This in turn locks down the ability for further deception in higher ToM(3) evident in Figure 3H), hinting that, at least in settings similar to ours, higher ToM benefits are asymptotic. We note that these dynamics are highly affected by the conservative pricing policy adopted by the ToM(2) seller as a precautionary mechanism against the ToM(1) deception. In turn, the ToM(3) is cornered, hence its policy resembles the ToM(1)’s policy.

One crucial limitation for the biological plausibility of our simulations is the high computational cost of deep recursive reasoning. This recursion forces a ToM(*k*) agent to solve |*M*|^*k*^ POMDP problems where *M* is the number of possible agent models. In Gmytrasiewicz and Doshi (2005) this complexity is evaluated to be PSPACE-hard for finite time horizon. From both computational complexity as well as from a bounded rationality perspective, this imply that the optimal policy of higher ToM levels is practically infeasible to compute in an accurate and timely manner. Indeed, previous theoretical work limited the highest ToM level to, in our nomenclature ToM(2) (for example, Adhikari & Gmytrasiewicz, 2021; Devaine et al., 2014; Goodie et al., 2012). Future work on efficient recursive beliefs representation is needed to make this problem computationally feasible.

While we highlighted the benefits of higher ToM levels, high ToM can also be detrimental. Of particular interest are misplaced high levels of recursion that may lead agents to be unnecessarily skeptical, when they are in fact faced with naïve agents. This would arise from our *k-level* cognitive hierarchy model where ToM(*k*) always strictly interpret actions as coming from the less sophisticated agent that is specifically ToM(*k* − 1)). Alon et al. (2023) investigated equivalent IPOMDP agents in a dynamic iterated game, showing deception and skepticism similar to the present work, but showing how misplaced high theory of mind gives rise to agents that stop trusting each other and lose reward in the process.

### Further Work

Our work leaves much opportunity for further investigation: First, a key factor of these deceptive dynamics is full observability of the actions of the players. Hence, future work might explore the information-seeking perspective of this model (Schulz et al., 2023), asking how much the buyer is willing to pay, or be paid, to disclose its actions and how much the seller is willing to pay to uncover the buyer’s action.

Secondly, certain settings of our task—like the extreme corners of the maze—encourage more or less deceptive behaviours. Future work might thus treat the settings of the task as an endogenous variable that the seller can control as part of its utility maximization planning. Naturally, in such a setting, buyers should have the option to avoid the shopping task altogether. We note that the control of the decision environment has links to the game theoretic work on preference elicitation and mechanism design (Becker et al., 1964; Roth, 2008).

Finally, potential future work could adopt a macroeconomic perspective and explore the multi-seller, multi-buyer case. In this setting, sellers need to make inferences about the actions of their competitors as well as about the preferences of their clients. In turn, the buyers’ planning might include a more sophisticated price query policy.

### Ethical and AI Alignment Concerns

Our work also offers words of caution for systems with theory of mind, particularly as the latter’s existence is currently heavily debated with regards to large language models (Kosinski, 2023; Sap et al., 2023; Ullman, 2023). These debates mainly center around whether LLMs possess what we would at most consider ToM(0), making relatively straightforward inferences about the mental state of others but not using them for planning in (semi-)competitive scenarios. LLM behavior has also been studied in more competitive game theoretic settings (Guo, 2023). For example Akata et al. (2023) investigated the behavior of LLMs in repeated games. Their results show how theory of mind like prompts can improve coordination and that GPT-4 has a tendency to play in an unforgiving manner. LLMs have also been coupled with more explicit planning strategies that share similarities with ours, for example for gameplay in Diplomacy (Meta Fundamental AI Research Diplomacy Team et al., 2022). We note however that ‘Cicero’ is explicitly barred from lying and deceit.

Our work explicitly shows how theory of mind can—without further handcrafting—give rise to deceitful and skeptical agents in a symbolic negotiation task. We note how our setting is a minimal representation of many semi-adversarial human-AI interactions, for example in recommender systems, dynamical pricing or human-LLM conversations. For the last of these, the emergence of theory of mind capabilities will have to be carefully monitored to understand what the LLMs reveal about their state of knowledge and goals, and how they interpret what other agents tell them.

A crucial aspect of the safety and ethics perspective on our results is that it is not theory of mind alone that gives rise to deception and skepticism. Rather, what produces this behavior is the coupling of theory of mind with a value function that mis-aligns the utilites of two agents. Our work therefore speaks to the alignment problem in AI safety. For example, the deceitful hiding of intentions may be crucial for the off-switch game (Hadfield-Menell et al., 2017).

## ACKNOWLEDGMENTS

We would like to thank Rahul Bhui and Stefan Bucher for helpful comments. This research was been partly funded by Israel Science Foundation grant #1340/18 (NA; JSR), by the Max Planck Society (NA, LS, PD) and the Humboldt Foundation (PD). PD is a member of the Machine Learning Cluster of Excellence, EXC number 2064/1 – Project number 39072764.

## Note

^1^ We here present a case that uses flat priors throughout, particularly with regards to the initial beliefs (*p*(*r*)) that the seller holds about the buyer’s preferences. Naturally, our paradigm would also allow for those priors to not be uniform but rather already be endowed with a first guess. Indeed, this raises the question as to whether our KL-Divergence metric is still appropriate in cases when the likelihood the seller receives aligns with its initial prior. In this case, the mean of the posterior belief of the seller would not shift. However, this reinforcement of the prior would still result in a reduction of the variance of the belief (because the seller receives an additional sample and can now be more certain about its estimate). In this case, the KL-Distance *D*_*KL*_(*p*(*r*|*a*_1_)‖*p*(*r*)) would still be above 0, indicating that the seller takes the buyers actions into consideration. In contrast, when the seller knows (through theory of mind), that the buyer is sending an uninformative signal, then it would simply keep it’s prior mean *and* variance, resulting in *D*_*KL*_(*p*(*r*|*a*_1_)‖*p*(*r*)) = 0, just as we showed before—even when the likelihood might superficially ’agree’ with the prior to a naïve receiver.
